# Effect of breastfeeding on the risk of breast cancer: a meta-analysis of observational studies

**DOI:** 10.1186/s13006-025-00796-4

**Published:** 2026-01-23

**Authors:** Rini Mutahar, Asri C. Adisasmita, Ratna Djuwita, Denni Joko Purwanto, Rini Anggraini, Yeni, Danny K. Aerosta

**Affiliations:** 1 Faculty of Public Health, Universitas Sriwijaya, Palembang, South Sumatra Indonesia; 2 Department of Epidemiology, Faculty of Public Health, Universitas Indonesia, Depok, Indonesia; 3 Surgical Oncology Department, Dharmais National Cancer Hospital, Jakarta, Indonesia

**Keywords:** Meta-analysis, Breastfeeding, Breast cancer, Cancer prevention, Lactation, Duration of breastfeeding, Case-control study

## Abstract

**Background:**

Breast cancer is the most common cancer among women worldwide, with its incidence increasing, particularly in low- and middle-income countries. Breastfeeding has been proposed as a modifiable protective factor, but conflicting evidence exists regarding the relationship between breastfeeding duration and breast cancer risk, warranting further investigation.

**Methods:**

This meta-analysis, conducted according to the PRISMA guidelines, included observational studies published in English between March 2014 and April 2024 and focused on case‒control and cohort studies. Literature searches were conducted via the EBSCO, PubMed, and Scopus databases. Studies were selected on the basis of the availability of odds ratios (ORs) or relative risks (RRs) with 95% confidence intervals (CIs), specifically analyses of the association between breastfeeding duration and breast cancer risk.

**Results:**

A total of 23 case‒control studies were included in the analysis. Never breastfeeding was associated with a significantly increased risk of breast cancer (OR: 1.40; 95% CI: 1.14, 1.72; I² = 84%). Breastfeeding for less than 12 months was associated with an even greater risk (OR: 3.59; 95% CI: 2.50, 5.18; I² = 31%) than breastfeeding for more than 11 months. Sensitivity analysis excluding three studies with inverse effects reduced heterogeneity (I² = 48%) and yielded a stronger association (OR: 1.69; 95% CI: 1.49, 1.91).

**Conclusion:**

This meta-analysis supports the protective effect of extended breastfeeding against breast cancer, particularly for durations exceeding 11 months. These findings underscore the importance of promoting breastfeeding as an accessible and cost-effective preventive strategy, particularly in resource-constrained settings.

**Supplementary Information:**

The online version contains supplementary material available at 10.1186/s13006-025-00796-4.

## Background

Breast cancer is the most prevalent form of cancer among women worldwide and presents a significant public health challenge. In 2020, breast cancer accounted for 685,000 deaths, making it the leading cause of cancer-related mortality among women, with approximately 2.26 million new cases reported that year [1, 2]. These alarming statistics underscore the urgent need for effective screening and prevention programs, particularly in developing countries where the incidence of breast cancer is expected to rise significantly in the coming years.

The increasing prevalence of breast cancer can be attributed in part to advancements in detection methods, especially in countries with well-established screening programs. While these improvements have led to earlier diagnoses and better prognoses in high-income countries, breast cancer continues to pose a substantial burden, especially in low- and middle-income countries where access to both screening and treatment is limited. Additionally, socioeconomic disparities further exacerbate breast cancer outcomes, with research highlighting urban‒rural disparities in disease prognosis, as seen in countries such as China [3].

Among various preventive approaches, hormonal and reproductive factors—particularly breastfeeding—have received considerable attention for their potential protective role against breast cancer. Physiologically, lactation suppresses ovulation and reduces cumulative lifetime exposure to estrogen, a well-established determinant of breast carcinogenesis. The differentiation of breast tissue and the increased turnover of epithelial cells during lactation may further decrease the likelihood of malignant transformation [4, 5]. Evidence from multiple studies supports these biological mechanisms, showing that breastfeeding is associated with a significantly lower risk of breast cancer, independent of parity [6, 7]. This association is believed to result from hormonal regulation, elevated prolactin levels, and structural changes in breast tissue during lactation, all of which may reduce susceptibility to carcinogenesis.

The duration of breastfeeding appears to further influence its protective potential. Studies suggest that each additional 12 months of breastfeeding is associated with a 43% reduction in breast cancer risk [8]. Evidence also indicates that prolonged breastfeeding (≥ 24 months) may further lower risk, though the protective effect may plateau at longer durations [9]. This may be due to extended periods of ovulation suppression and sustained hormonal changes. Despite these findings, some studies have failed to observe a significant association between breastfeeding and breast cancer risk. For instance, research in Iran found no correlation between breastfeeding duration and breast cancer, possibly due to genetic or regional factors [10]. Similarly, a study from Cyprus identified an inverse relationship but could not confirm a clear dose-response pattern.

Despite the substantial body of evidence supporting the protective effects of breastfeeding, inconsistencies remain across populations and study designs. These variations highlight the need for a comprehensive synthesis of existing data to determine the strength and consistency of this association. This systematic review differentiates itself by focusing on breastfeeding duration and its relation to breast cancer risk. While Zhou et al. [11] compared breastfeeding vs. never breastfeeding and shortest vs. longest durations, they did not detail breastfeeding duration further. Unar-Munguía et al. [12] emphasized exclusive breastfeeding, whereas our review focuses on overall breastfeeding duration without distinguishing modes. Unar-Munguía et al. [12] also conducted subgroup analyses based on menopausal status and parity, while Oikonomou et al. [13] provided a narrative review without pooled estimates. This meta-analysis includes studies published from 2014 to 2024, incorporates data from underrepresented regions, and applies sensitivity analyses for more robust and relevant findings. Therefore, this study aims to systematically evaluate the relationship between breastfeeding and breast cancer risk through a meta-analysis of observational studies, with a focus on breastfeeding duration and potential sources of heterogeneity.

## Methods

This study was conducted in accordance with the Preferred Reporting Items for Systematic Review and Meta-Analysis (PRISMA) guidelines [14]. The review has been registered in the International Prospective Register of Systematic Reviews (PROSPERO) under registration number CRD42024532351.

### Data sources and searches

A comprehensive search for epidemiological studies was conducted through the Scopus, PubMed, and EBSCO databases. The search terms and keywords used are listed in Table 1. Eligible studies included observational designs such as case‒control and cohort studies. Only English-language publications published between March 2014 and April 2024 were considered.

**Table 1:** Detailed electronic search strategy for the Scopus, PubMed, and EBSCO databases

Database	The detailed electronic searching strategy
Scopus	(TITLE-ABS-KEY (“breast neoplasms” OR “breast cancer” OR “breast carcinoma” OR “breast tumor” OR “breast malignant”)) AND (TITLE-ABS-KEY (“breastfeeding*” OR “lactation*” OR “suckling*” OR “breast milk”)) AND (TITLE-ABS-KEY (“cohort study” OR “observational study” OR “case control” OR “cross sectional” OR “longitude”))
Pubmed Ebsco	(“breast neoplasms” [MeSH] OR “breast cancer” OR “breast carcinoma” OR “breast tumor” OR “breast malignant”) AND (breastfeeding OR lactation OR suckling OR “breast milk” OR lactating OR breastfed OR “breast feeding” OR “breast-feeding”) AND (“cohort study” OR “observational study” OR “case control” OR “cross sectional" OR longitude)

### Study selection and data extraction

Studies were included in the analysis if they met the following criteria: (1) they employed a case‒control or cohort design; (2) the outcome of interest was newly diagnosed breast cancer cases (recurrent cases were excluded), verified by pathological biopsy or other standard diagnostic methods, (3) studies that exclusively reported invasive breast cancer were not excluded they included all types of breast cancer, whether in situ or invasive; and (4) they reported relative risks (RRs), hazard ratios (HRs), or odds ratios (ORs) with 95% confidence intervals (CIs) for the lowest versus highest breastfeeding duration categories.

The study selection process was conducted in two stages. The first screening of titles and abstracts was independently carried out by two authors (DK and RA). The full-text articles were then reviewed by two other authors (RM and YN). In cases of disagreement, a consensus was reached through discussion. If consensus was not achieved, a third author (AA for the first screening, DJ for the second) provided the deciding opinion on study eligibility.

Data extraction followed a standardized approach via a predefined worksheet. Full-text articles were included if the complete manuscript could be obtained through any legal means (institutional subscription, author contact, interlibrary loan, or open-access download). The extracted data included the first author’s surname, year of publication, study location, study design (cohort or case–control), sample size (cohort cases and incidence rates for cohort studies, or case and control numbers for case–control studies), exposure variables (e.g., ever breastfeeding vs. never breastfeeding, breastfeeding duration > 1 year vs. <1 year), outcome variables (breast cancer incidence), risk measures (OR or HR/RR with 95% CI), and statistical adjustment variables.

### Study quality assessment

The quality of the included studies was independently assessed by two authors (RM and RA) via the Newcastle‒Ottawa Quality Assessment Scale (NOS) for observational studies [15]. The NOS evaluates three domains: (1) selection of study groups (four items); (2) comparability of cases and controls/cohorts on the basis of design or analysis (one item, worth up to two stars); and (3) exposure ascertainment for case‒control studies or outcome assessment for cohort studies (three items). Discrepancies in the quality assessments were resolved through discussion or consensus. If necessary, a third author (RD) was consulted to resolve disagreements. The quality assessment aimed to rank the methodological rigor of each study rather than exclude studies from the analysis.

### Data synthesis and analysis

For each outcome, a meta-analysis was performed via RevMan 5.3 software if more than two studies were available. The meta-analysis included dichotomous data, with results reported separately on the basis of RRs/HRs for cohort studies and ORs for case‒control studies, along with their respective 95% CIs. All association measures used in the analysis were adjusted estimates. This study employed the “ever breastfeeding” group as the reference category for estimating the odds ratio (OR) of breast cancer risk. In instances where a study used a different reference group—such as “never breastfeeding”—the reported OR values were converted accordingly. Similarly, for the subgroup analysis assessing the association between the duration of breastfeeding and breast cancer risk, the reference group was defined as women who had breastfed for more than 11 months. The selection of the breastfeeding duration cutoff of < 12 months and > 11 months was made for both practical and clinical reasons. This threshold was chosen because many studies report findings related to breastfeeding duration using 12 months as the cutoff. Clinically, breastfeeding for more than 11–12 months is often considered to provide a stronger protective effect against breast cancer risk, based on evidence showing a dose-response relationship between breastfeeding duration and a reduction in breast cancer risk [9].

The primary analyses were conducted using a random-effects model to calculate the overall RR/OR of breast cancer, as between-study heterogeneity was high in most associations evaluated in the meta-analysis. To assess heterogeneity among studies, we used Cochran’s Q test and the I^2^ statistic, considering I^2^ values of > 50% as high heterogeneity.

To find sources of heterogeneity and assess results in each subgroup of the included studies, we performed subgroup analyses on the basis of source of control (hospital-based vs. population-based), participant (all women vs. parous women), and study quality (good vs. fair). These variables were selected on the basis of the importance of subgroups for assessing our results and their effects on between-study heterogeneity. We also conducted a sensitivity analysis via a random effects model, in which each study was excluded to examine the influence of that study on the overall estimate. Publication bias was evaluated visually via a funnel plot.

## Results

**Fig. 1:**
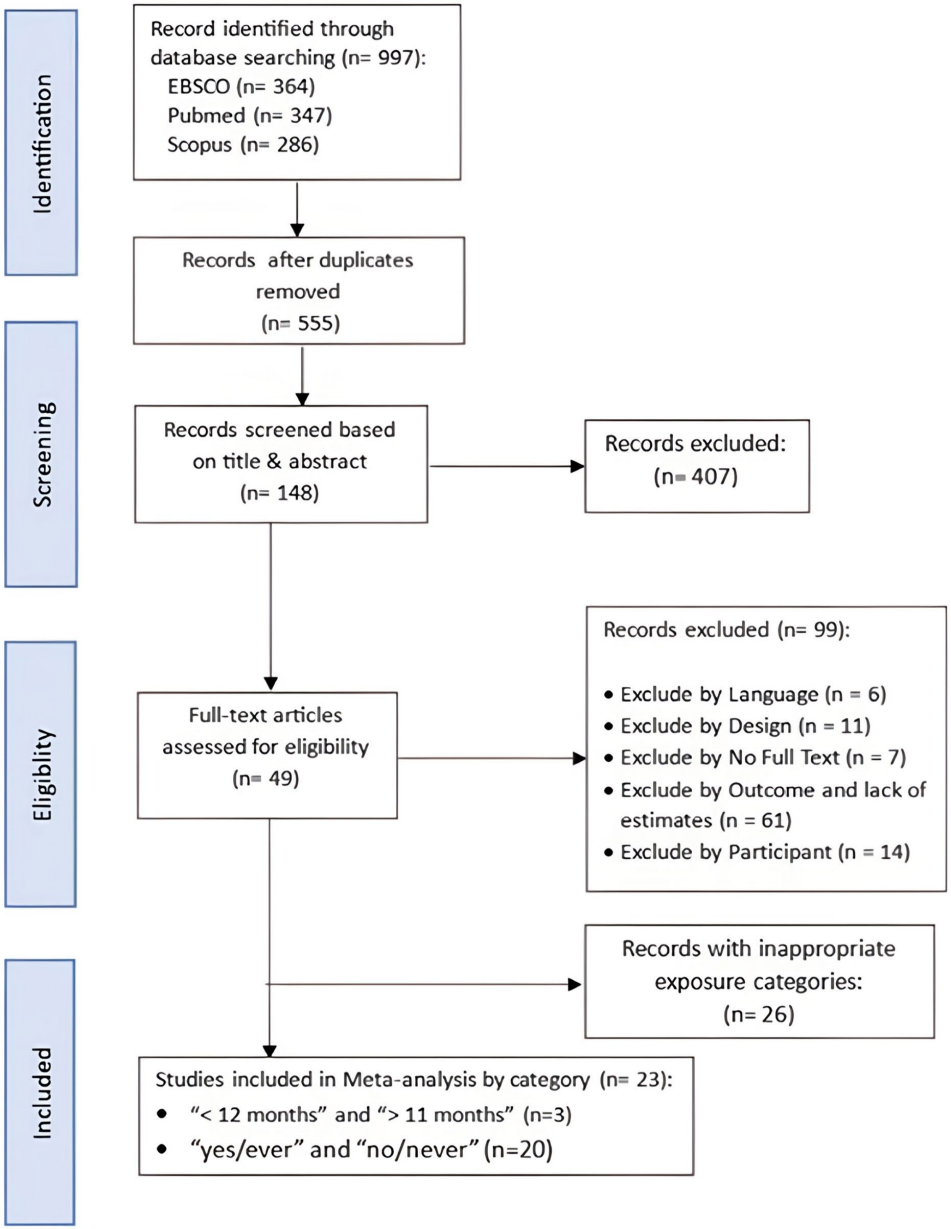
Flow diagram of exclusions

Figure 1 provides a flow diagram summarizing the study selection process for this meta-analysis. The initial database search across EBSCO (*n* = 364), PubMed (*n* = 347), and Scopus (*n* = 286) produced a total of 997 records. After removing duplicates, 555 records remained, which were screened on the basis of their titles and abstracts. During this phase, 407 studies were excluded because they did not meet the inclusion criteria. Notably, no cohort studies were identified.

Among the remaining 148 full-text articles assessed for eligibility, 99 were excluded for the following reasons: language incompatibility (*n* = 6), inappropriate study design (*n* = 11), unavailability of full-text articles (*n* = 7), lack of extractable outcomes or adjusted estimates (*n* = 61), and insufficient description of participant inclusion or exclusion criteria (*n* = 14). As shown in the newly added exclusion box in Figs. 1 and 26 studies were excluded for the use of inappropriate exposure categories that could not be harmonized with our reference definitions (e.g., comparisons other than “ever” vs. “never” or “< 12 months” vs. “> 11 months”). Ultimately, 23 case‒control studies were included in the meta-analysis: three assessed breastfeeding duration (“< 12 months” vs. “> 11 months”) [16–18], and 20 compared “ever” vs. “never” [19–38].

Table 2 outlines the key characteristics of the included studies, which were conducted in diverse countries such as Iraq, Indonesia, Ethiopia, and China. The studies employed both hospital-based and population-based case‒control designs, with participants aged 18–80 years. Sample sizes varied significantly, ranging from 100 cases and controls in Sukma (2021) to 25,343 participants in Jeong (2017) [16, 35]. Each study considered important confounding variables such as age at menarche, contraceptive use, and parity. The association between breastfeeding and breast cancer risk was measured via odds ratios (ORs) and 95% confidence intervals (CIs).

**Table 2:** Study characteristics of the studies included in the meta-analysis (*n* = 23)

No.	First author, year	Country	Study design^a^	Age^b^	Participant	Sample size (cases/ control)^c^	Exposure reference	Confounding variables	Adjusted OR	CI 95%	
1	Ramadan, 2023 [17]	Iraq	Case‒control (hospital based)	35–65	All women	121/484	> 11 months	Confounding by age of menarche, first pregnancy age, contraceptive pill usage, history of abortion, birth history, age of menopause, hormone replacement therapy, body mass index	1.84	0.79, 4.38	
2	Sukma, 2021 [16]	Indonesia	Case‒control (hospital based)	-	All women	100/100	≥ 12 months	Confounding by childbirth age of first child	3.66	1.64, 8.16	
3	Fentie, 2023 [18]	Ethiopia	Case‒control (hospital based)	18–50	All women	135/73	> 1 year	Confounding by age, residence, marital status, educational, occupation, monthly income, dietary patterns, number of children ever born, age at first birth, menopause, chest radiation therapy, age of menarche, use modern of contraceptive, physical exercise status	4.33	2.78, 6.89	
4	Romie, 2018 [19]	4 Latin American Countries (Chile, Colombia, Costa Rica, And Mexico)	Matched case‒control (population-based)	30–44	Parous women	213/252	Never	Matched by age (± 3 years), city district of residence, and health insurance institution. Confounding by age, country, city district, health insurance institution, education level, history of benign breast disease, physical activity, and waist circumference when appropriate.	0.56	0.34, 0.93	
5	Yang, 2015 [20]	China	Matched case control (hospital based)	22–71	All women	112/139	No	Matched by age (± 5 years). Confounding by age, menarche age, menopause, menopause age, fertility, parity, age at first parity, abortion, abortion times, marriage, body mass index	0.827	0.411, 1.663	
6	Tan, 2018 [21]	Malaysia	Case control (hospital based)	40–74	Parous women	1474/3198	Never	Confounding by age, ethnicity, and hospital, education, except history of breast surgery.	0.56	0.48, 0.65	
7	Wahidin, 2018 [22]	Indonesia	Case control (hospital based)	≥ 15	All women	381/381	No	Confounding by oral contraceptive use, age, unhealthy diet, history of benign breast tumor, hospital	1.83	1.23, 2.72	
8	Xie, 2022 [23]	China	Matched case control (hospital based)	25–70	All women	1170/1170	Yes	Matched by age (± 3 years). Confounding by parity.	1.543	1.02, 2.33	
9	Aich, Ranen Kanti, 2016 [24]	India	Matched case control (population-based)	19–80	All women	1463/1440	No	Matched by socioeconomic, environmental, racial, and ethnicity. Confounding by age, age at menarche, educational status, menstrual status, breastfeeding, family history of breast cancer, family history of ovarian cancer, first pregnancy outcome, body mass index, average duration lactation, age at first full term pregnancy.	0.805	0.480, 1.349	
10	Al-Amri, 2015 [25]	Saudi Arabia	Matched case control (population based)	30–69	All women	58/290	No	Matched by age (± 5 years). Confounding by age at marriage, number of pregnancies, age at menopause, family history of breast cancer, and history of radiation.	0.3	0.13, 0.69	
11	Bashamakha, 2019 [26]	Yemen	Matched case control (hospital based)	< 50 - >50	Parous women	91/198	Yes	Matched by age (± 5 years), District residency, and year of diagnosis. Confounding by marital status, hypertension, family history of malignancy, menopause	1.7	1.1, 5	
12	Beg, 2023 [27]	Pakistan	Matched case control (population based)	35,6–58,8	All women	161/149	No	Matched by demographic characteristics.	3.315	2.094, 5.249	
13	Holm, 2017 [28]	Sweden	Case control (population based)	25–88	Parous women	2069/13,950	Yes	Confounding by born in sweden or not, age, education level, parity, age at first birth, and body mass index	1.59	1.23, 2.03	
14	Galukande, 2016 [29]	Uganda	Case control (hospital based)	< 50 and > 50	All women	111/237	No	Confounding by coc (combined oral contraceptive) use, age, residence, alcohol, parity, afb (late age at first full-term birth), menarche	0.04	0.01, 0.18	
15	Duche, 2021 [30]	Ethiopia	Case control (hospital based)	> 15	All women	110/110	Yes	Confounding by age, physical activity, parity, abortion, menopausal status. Oral contraceptive use, body mass index	3.4	1.2, 9.67	
16	Bustamante-Montes, 2019 [31]	Mexico	Matched case control (hospital based)	41,96 − 64,04	All women	101/101	No	Matched by age (± 5 years) and residence. Confounding by age, type of health insurance, residental status	0.12	0.02, 0.60	
17	Hosseinzadeh, 2014 [32]	Iran	Matched case control (hospital based)	36,4–58,3	All women	140/280	No	Matched by age. Confounding by educational level, menopausal status, a high-fat diet, abortion, passive smoking, oral contraceptive pill use, stress, migration, fruit and vegetables eat	0.39	0.16, 0.97	
18	Ichida, 2015 [33]	Japan	Case control (hospital based)	20–69	Parous women	17/1001	Never (bottle only)	Confounding by age, oral contraceptive use, menopausal status, parity, parity and breastfeeding, family history of breast cancer	0.86	0.30, 2.49	
19	Ilic, 2015 [34]	Serbia	Matched case control (hospital based)	35–80	Parous women	168/171	Never	Matched by age (± 2 years), time of hospital admittance, and place of residence (rural or urban). Confounding by age, place of residence, educational level, employment, age at menarche, oral contraceptive use, number of pregnancies, number of live births, age at first birth, menopausal status, abortion history, family history of breast cancer, body mass index, alcohol use, tobacco use;	2.9	1.02, 8.22	
20	Jeong, 2017 [35]	Korea	Matched case control (population based)	40–74	All women	12,598/12,745	Never (never + no child)	Matched by age and year of enrollment. Confounding by age, family history of breast cancer, age at menarche, age at first full-term pregnancy, duration of oral contraceptive use, body mass index, and number of childbirths	0.6	0.55, 0.64	
21	Leon Guerrero, 2017 [36]	Mariana Islands (US)	Matched case control (hospital based)	25–80	Parous women	93/166	No	Matched by age, ethnicity, and location (saipan or guam). Confounding by age at reference and ethnicity	1.06	0.58, 1.93	
22	Lin, 2019 [37]	China	Matched case control (hospital based)	50,76 − 11,73	All women	1215/1195	No	Matched by age Confounding by abortion history, family history of malignant tumor, and history of benign breast surgery	0.68	0.56, 0.81	
23	Nishiyama, 2020 [38]	Japan	Case control (population based)	22,91	All women	530/1043	No	Confounding by age	0.64	0.50, 0.81	

a. For hospital-based studies, controls were recruited from non-cancer patients or hospital visitors at the same hospitals, as described in the original articles

b. Age refers to the age range of participants at study entry

c. “Cases” refers to participants diagnosed with breast cancer, and “Controls” refers to participants without breast cancer

The Newcastle‒Ottawa Scale (NOS) was used to assess the quality of the included studies, as detailed in Supplementary Table 1.

**Fig. 2:**
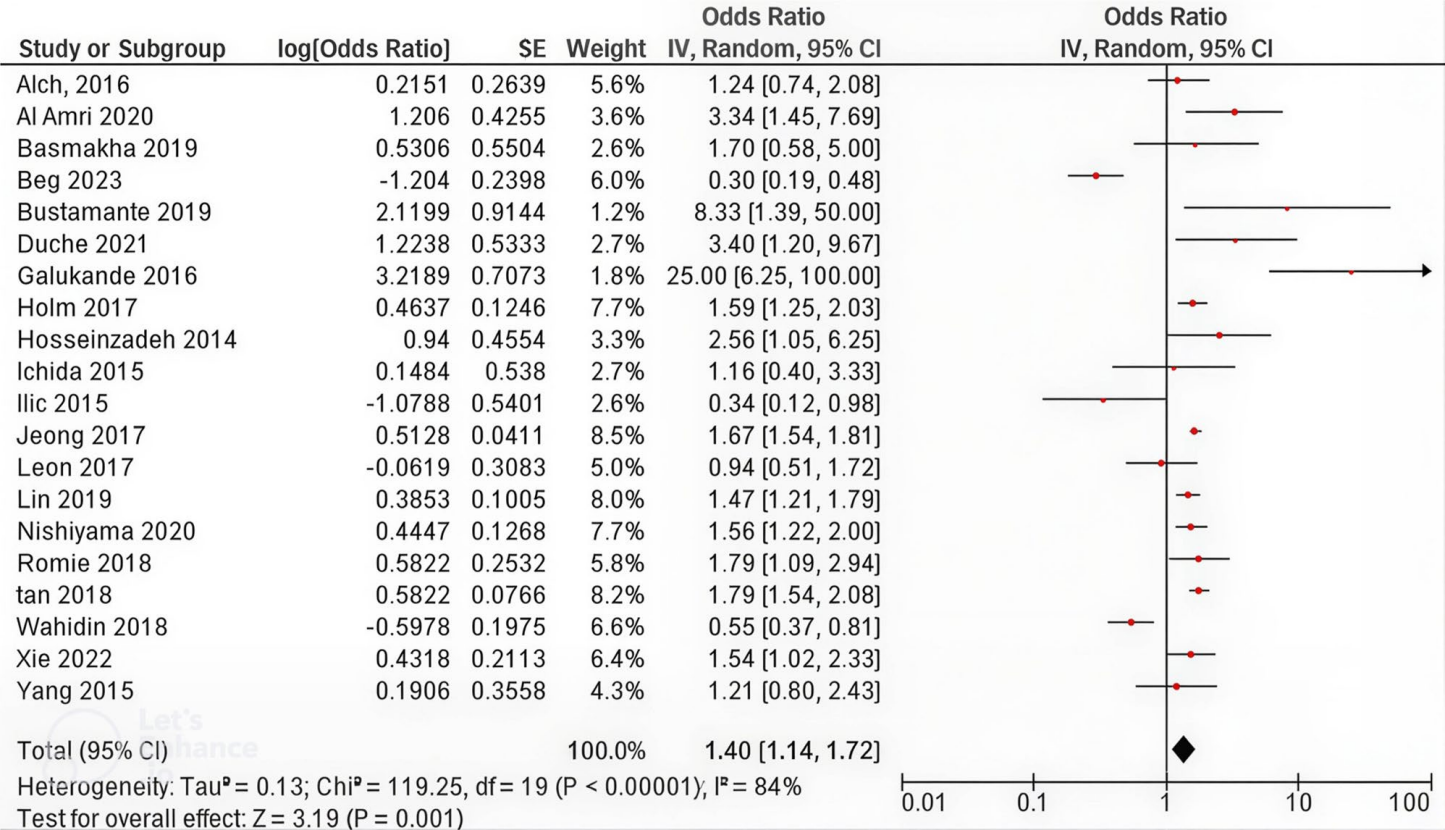
Summary of the meta-analysis of the effects of breastfeeding on the risk of breast cancer (breastfeeding Never vs. Ever)

**Fig. 3:**
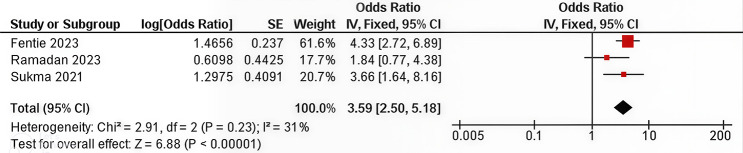
Summary of the meta-analysis of the effects of breastfeeding on the risk of breast cancer (breastfeeding < 12 months vs. > 11 months)

Figure 2, a random-effects model revealed that the pooled odds ratio was 1.40 (95% CI: 1.14, 1.72), indicating that women who have never breastfed have a 40% higher risk of developing breast cancer than those who have breastfed. The heterogeneity analysis yielded χ² = 119,25 (*p* < 0.00001) and I² = 84%, reflecting high variability. Figure 3 shows the meta-analysis of the three case‒control studies that assessed breastfeeding duration. The pooled OR was 3.59 (95% CI 2.50, 5.18), indicating that breastfeeding for < 12 months was associated with a 3.59-fold greater risk of breast cancer than breastfeeding for > 11 months. Heterogeneity for this comparison was low (χ² = 2.91, *p* = 0.23; I² = 31%).

**Fig. 4:**
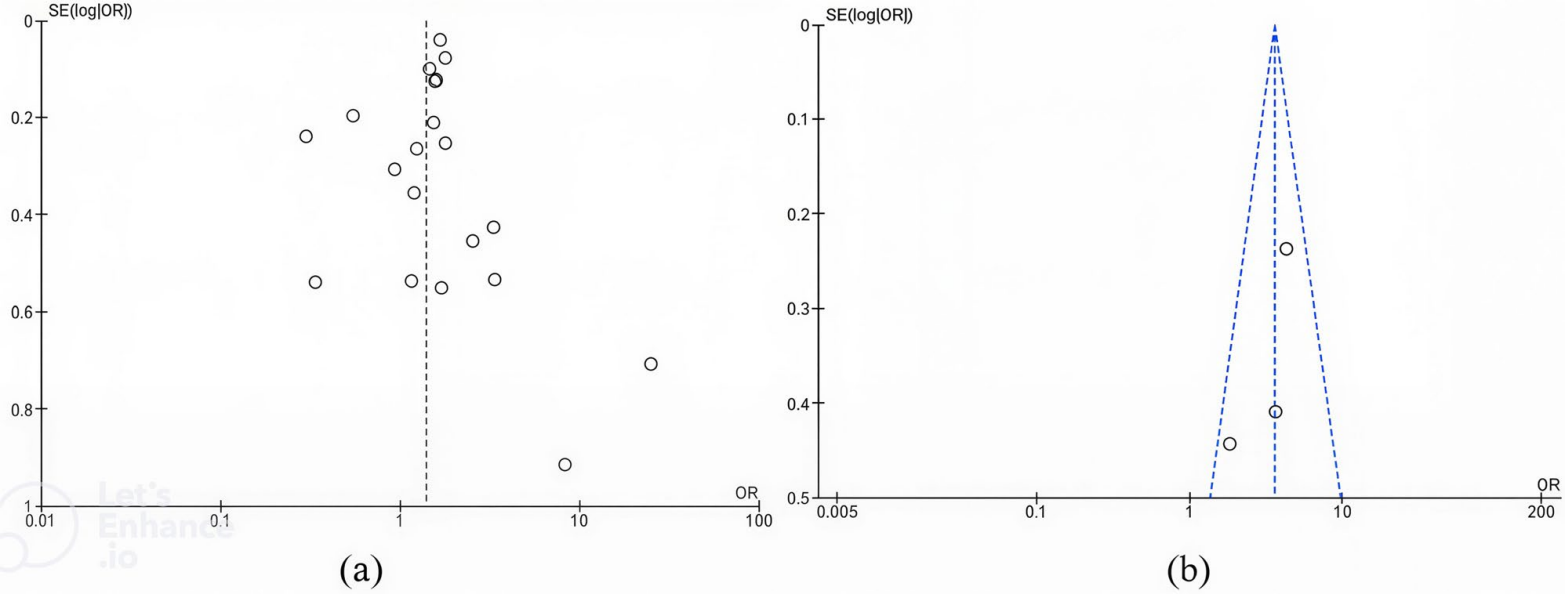
Funnel plot of standard error by point estimate for assessment of publication bias. Studies that evaluated the effect of breastfeeding on breast cancer: (**a**) breastfeeding never vs. ever; (**b**) breastfeeding < 12 months vs. > 11 months

The funnel plot in Fig. 4 assesses potential publication bias in this meta-analysis. Overall, Panel A shows some asymmetry, which may indicate the presence of publication bias or small-study effects in the comparison of never versus ever breastfeeding. In contrast, Panel B demonstrates a relatively symmetrical distribution of studies, suggesting a low risk of publication bias for the analysis comparing breastfeeding durations (< 12 months vs. > 11 months). While funnel plots do not directly assess heterogeneity, such asymmetry could also reflect underlying methodological or population differences across studies.

## Discussion

This meta-analysis of 23 case‒control studies demonstrated that never breastfeeding is associated with a 40% higher risk of breast cancer, whereas breastfeeding for < 12 months confers a 3.59-fold greater risk relative to > 11 months of lactation. Overall, the study demonstrated that any history of breastfeeding is tied to a lower chance of developing breast cancer than never breastfeeding. Moreover, longer durations strengthened this protective effect. The findings of our study are consistent with those of previous meta-analyses. Zhou et al. [11] demonstrated an inverse relationship between breastfeeding and breast cancer risk, with a relative risk (RR) of 0.613 (95% CI: 0.442, 0.850) for ever breastfeeding compared to never breastfeeding, and an even stronger association for longer breastfeeding durations, with an RR of 0.471 (95% CI: 0.368, 0.602) for the longest durations. Similarly, Unar-Munguía et al. [12] reported an SRR of 0.72 (95% CI: 0.58, 0.90) for exclusive breastfeeding, indicating a stronger protective effect compared to non-breastfeeding. Their findings suggest that exclusive breastfeeding offers more substantial protection due to its hormonal effects. Furthermore, Oikonomou et al. [13] highlighted that breastfeeding, especially for durations beyond one year, was consistently associated with a reduced risk of breast cancer. Our study also found that longer breastfeeding durations, particularly beyond 12 months, provided stronger protection, which aligns with the conclusions drawn by these studies, emphasizing the importance of extended breastfeeding as a preventive measure against breast cancer.

Several mechanisms seek to elucidate the inverse relationship between breastfeeding and breast cancer risk. A significant explanation is hormonal changes during lactation, especially a reduction in ovulatory cycles, which leads to less total exposure to estrogen, a hormone intimately connected to the beginning of breast cancer [39]. Extended periods of anovulation during lactation result in less lifetime exposure to estrogen and progesterone, which is especially important in reducing the frequency of hormone receptor-positive breast cancers [40]. Similarly, breastfeeding increases prolactin levels, making breast tissue maturation less susceptible to mutations [41]. Another proposed mechanism is that breastfeeding aids in removing potential carcinogens from breast tissue through mechanical processes, facilitating the turnover of breast epithelial cells, which may eliminate cells that could turn malignant [5, 42]. Furthermore, slow involution of breast tissue during and following prolonged breastfeeding may offer long-term protection, whereas sudden involution in women who do not breastfeed may not provide this advantage [37]. This preventive function is more apparent in specific subtypes, such as triple-negative breast cancer, which exhibit more pronounced inverse correlations with longer breastfeeding durations [43].

Although these biologically plausible pathways exist, it is necessary to recognize their methodological limitations. This meta-analysis included only case‒control studies, making the results susceptible to potential biases. Selection bias may arise if the controls are not sourced from the same underlying population as the cases. In contrast, recall bias is prevalent due to the retrospective collection of breastfeeding history, which may result in inaccurate reporting. If the accuracy of reported breastfeeding histories differs between cases and controls, differential misclassification could occur. The use of blinding in case‒control studies is a further issue. Blinding helps reduce bias in data interpretation by using data collectors or analysts unaware of the participants’ illness state. Many case‒control studies, however, do not clearly state whether blinding was used during data collection or analysis, hence increasing the possibility of biased reporting of associations.

In addition, heterogeneity is a frequent issue in implementing meta-analyses. The total research comparing women who did with those who never breastfed showed notable heterogeneity, as seen by an I² value of 84% and a pooled odds ratio (OR) of 1.40 (95% CI: 1.14, 1.72). This finding indicates significant variation among the included studies that cannot be solely ascribed to sample size or random factors. Several subgroup studies have been conducted to investigate the causes of this variation. Analyses taking into account research source (hospital-based vs. population-based), participant characteristics (all women vs. parous women), and study quality did not yield notable variations in terms of heterogeneity (*p* > 0.05). The findings show that the disparities in outcomes among the studies were not significantly affected by changes in research site, quality, or participant traits (Supplementary Figs. 2–4).

When three studies showing inverse effect directions (OR < 1), which would have added to the heterogeneity, a sensitivity analysis was performed to improve the robustness of the findings. With a pooled odds ratio of 1.69 (OR: 1.69; 95% CI: 1.49, 1.91) and a notably lower I² of 48%, this enhanced study yielded a more consistent and stronger link. These results highlight the main conclusion that extended breastfeeding offers a significant preventative advantage against breast cancer. After accounting for likely outlier studies with different effect directions, the sensitivity analysis verified the consistency and dependability of the associations. (Supplementary Fig. 5).

Despite these limitations, this meta-analysis has several strengths. With a significant sample size of more than 22,631 breast cancer patients and about 38,873 controls, this study increases the statistical power and precision of the effect estimates. Considering research from different geographic areas increases the generalizability of the findings. The meta-analysis examined each study’s age, menopausal status, contraceptive use, and lifestyle choices, among other confounding factors. Careful control for these factors increases the validity of the reported correlations and reduces the likelihood that the findings are influenced by bias or unreported confounders. The scope of the evidence included in this review reflects the predefined inclusion criteria, which may not capture some of the most recent or region-specific studies.

This meta-analysis revealed that breastfeeding, particularly for durations longer than 11 months, is linked to a lower risk of breast cancer. Sensitivity analysis confirmed the findings across several research populations by demonstrating lower heterogeneity and stronger effect estimates after eliminating studies indicating inverse relationships. Public health campaigns should prioritize breastfeeding, not only for its proven benefits to newborn health but also for its possible role in lowering breast cancer rates. Especially in low-resource communities where other cancer-preventive activities can be less accessible, breastfeeding can be a reasonable and economical approach to prevent cancer. To reduce the risk of breast cancer, prolonged nursing should be encouraged together with other preventive health measures.

## Conclusions

This meta-analysis revealed that breastfeeding, especially for periods longer than 11 months, is correlated with a decreased risk of breast cancer. The results were comparable across several research populations and were further validated by sensitivity analyses, which revealed decreased heterogeneity and more robust effect estimates after excluding studies showing inverse connections. Public health initiatives should prioritize the promotion of breastfeeding, not only for its established advantages in terms of infant health but also for its potential to reduce the incidence of breast cancer. Breastfeeding can be a practical and affordable way to prevent cancer, especially in low-resource areas where other cancer-preventive actions may be less common. Extended breastfeeding should be promoted alongside other preventative health interventions to mitigate breast cancer risk.

## Supplementary Information

Below is the link to the electronic supplementary material.

Supplementary Material 1

## Data Availability

No datasets were generated or analysed during the current study.

## Declarations

### Generative AI and AI-assisted technologies in the writing process

During the preparation of this manuscript, the authors used ChatGPT (OpenAI) and Grammarly to improve language clarity, correct grammar, and refine sentence structure. Following the use of these tools, the authors reviewed and edited the content thoroughly and accept full responsibility for the integrity and accuracy of the final manuscript.
