# Supplementary material for: Effect of breastfeeding on the risk of breast cancer: a meta-analysis of observational studies
Source: Int Breastfeed J. 2026 Jan 23;21:23. doi: 10.1186/s13006-025-00796-4 (PMC12911000; doi:10.1186/s13006-025-00796-4)

**SUPPLEMENT**

Table 1. Result of the study quality

| Newcastle-Ottawa Quality Assessment Form for Case-Control Studies | | | | | |
| --- | --- | --- | --- | --- | --- |
| Quality^*^ | Total | Outcome | Comparability | Selection | Author (year) |
| Fair | 5 | 2 | 2 | 1 | Ramadan (2023) |
| Good | 7 | 2 | 2 | 3 | Sukma (2021) |
| Good | 7 | 2 | 2 | 3 | Fentie (2023) |
| Good | 7 | 2 | 2 | 3 | Romie (2018) |
| Good | 7 | 2 | 2 | 3 | Yang (2015) |
| Good | 7 | 2 | 2 | 3 | Tan (2018) |
| Good | 7 | 2 | 2 | 3 | Wahidin (2018) |
| Good | 7 | 2 | 2 | 3 | Xie (2022) |
| Good | 7 | 2 | 2 | 3 | Aich (2016) |
| Good | 7 | 2 | 2 | 3 | Al-Amri (2015) |
| Good | 7 | 2 | 2 | 3 | Bashamakha (2019) |
| Good | 8 | 2 | 2 | 4 | Beg (2023) |
| Good | 8 | 2 | 2 | 4 | Holm (2017) |
| Fair | 5 | 2 | 1 | 2 | Galukande (2016) |
| Fair | 6 | 2 | 1 | 3 | Duche (2021) |
| Good | 8 | 2 | 2 | 4 | Bustamante-Montes (2019) |
| Fair | 6 | 2 | 2 | 2 | Hosseinzadeh (2014) |
| Fair | 6 | 2 | 1 | 3 | Ichida (2015) |
| Good | 7 | 2 | 2 | 3 | Ilic (2015) |
| Good | 7 | 2 | 2 | 3 | Jeong (2017) |
| Good | 7 | 2 | 2 | 3 | Leon Guerrero (2017) |
| Good | 7 | 3 | 2 | 2 | Lin (2019) |
| Good | 7 | 2 | 1 | 4 | Nishiyama (2020) |

Table 2. Subgroup analysis based on hospital-based vs population-based


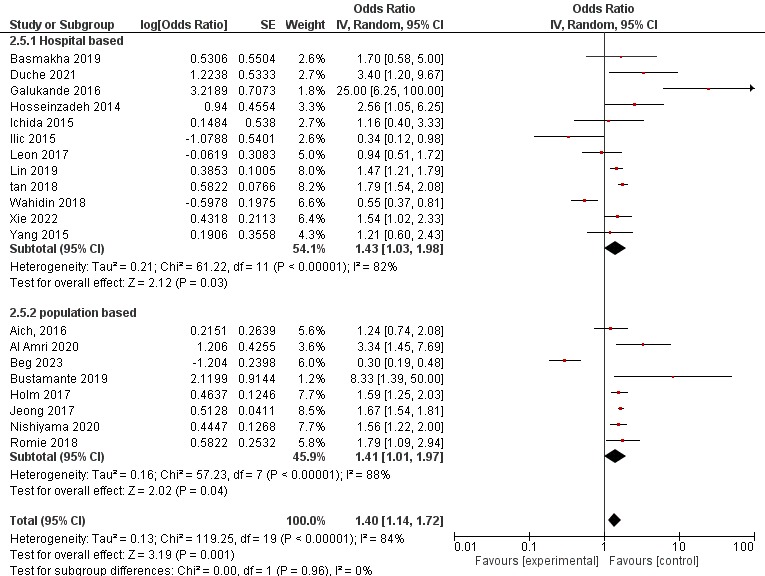


Table 3. Subgroup analysis based on all women vs parous women


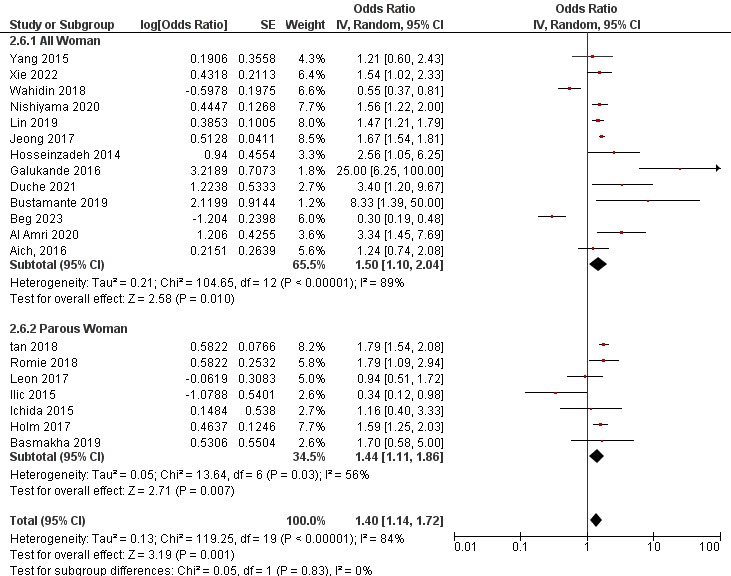


Table 4. Subgroup analysis based on study quality


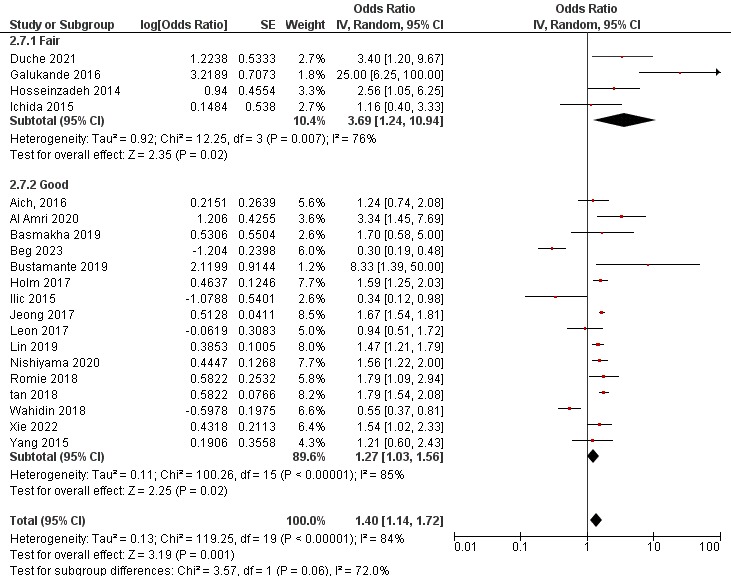


Table 5. Sensitivity analysis (excluded inverse associations between breastfeeding and breast cancer)


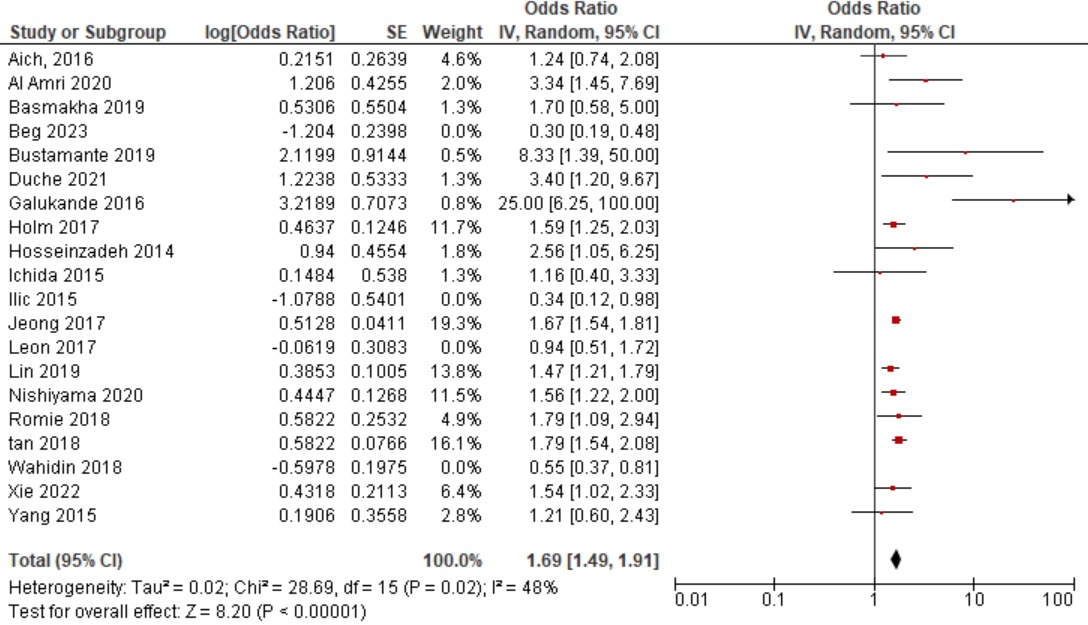

Supplement: Supplementary file 1 — Supplementary Material 1 [file 13006_2025_796_MOESM1_ESM.docx]
